# Association Between Head Circumference Growth and Peripheral Nerve Cross-Sectional Area Growth in Infants: A Potential Future Biomarker for Central and Peripheral Nerve Maturation

**DOI:** 10.1055/a-2747-7359

**Published:** 2025-11-28

**Authors:** Noé P. Bürke, Lynn Jansen, Erin West, Janina Wurster, Philip J. Broser

**Affiliations:** 1 Faculty of Medicine, University of Zurich, Zurich, Switzerland; 2 School of Medicine, University of St. Gallen, St. Gallen, Switzerland; 3 Clinical Trials Unit, HOCH Health Ostschweiz, Universitäres Lehr- und Forschungsspital, Sankt Gallen, Switzerland; 4 Children's Hospital of Eastern Switzerland, Sankt Gallen, Switzerland; 5 Medical Faculty, University of Basel, Basel, Switzerland

**Keywords:** neurodevelopment, nerve cross-sectional area, head circumference, ultrasound

## Abstract

**Objective:**

To analyze the association between the growth of the central and peripheral nervous systems (PNS) in children aged 0 to 3 years.

**Method:**

A total of 40 participants were included in this cross-sectional study to analyze the association between the growth and development of the peripheral and central nervous system (CNS). Using high-resolution ultrasound, the cross-sectional area (CSA) of the median nerve was measured at three locations (wrist, forearm, and upper arm) representing the development of the PNS and then compared with the head circumference (HC) as a proxy for the CNS development.

**Results:**

There was a significant correlation between HC and the CSA of the median nerve at the three measured locations. When looking at adjusted linear regression models, HC appeared to be a stronger predictor of nerve CSA size than age.

**Conclusion:**

The observed association between nerve CSAs and HC growth indicates a parallel size increase. This association may have clinical relevance because both HC and nerve CSA could potentially serve as complementary markers for neurodevelopmental monitoring, that is, myelination, and may contribute to the early identification of atypical developmental patterns, though confirmatory longitudinal data are required.

## Introduction

In the first 2 years of life, children develop from dependent, immobile neonates into toddlers who can walk independently, explore their surroundings, and communicate verbally. For a child to achieve these milestones, the central nervous system (CNS) and the peripheral nervous system (PNS) must mature and undergo significant developmental processes. An important part of this maturation is the myelination of the axons in both the CNS and the PNS.

Myelination begins before birth at around the 4th month of fetal development. 1 Raimbault 2 found that the myelination of peripheral nerves leads to an increase in nerve conduction velocity (NCV). An almost doubling of the sensory and motor NCV was observed in the first 2 years of life (from 20–25 m/s in newborns to 45–55 m/s in 2-year-olds).

The myelination of the CNS also begins very early in fetal development, and by the end of the 2nd year of life, the brain is almost completely myelinated. 3 Knickmeyer 4 and Lebel 5 each observed a rapid increase in white matter in magnetic resonance imaging. This development is accompanied by an increase in intracranial volume and brain size, both of which correlate closely with head circumference (HC). 6 7 8 9

The nerve maturation process can also be observed in the PNS at the structural level, characterized by an increase in the nerve cross-sectional area (CSA) as a result of increased myelination. 10 These structural changes can be precisely analyzed using high-resolution ultrasound imaging. 11 12 13 Jenny et al 14 found that median nerve CSA growth followed a logarithmic pattern and suggested that a similar model could describe the increase in NCV. This parallel course of the increases in nerve CSA and NCV is also described in the study by Broser and Lütschg. 15

The graphical evaluation of the increase in NCV in the study by Broser and Lütschg 15 and the temporal rise in CSA in the study by Jenny et al 14 reveals a growth pattern resembling the percentile curves commonly used in pediatrics to track normal child development. Notably, one such parameter is HC, which follows a similar trajectory to these graphs and, as previously discussed, correlates strongly with an increase in intracranial volume and brain size.

These similarities raise the question of whether there is a correlation between median nerve CSA and HC and, in turn, a connection between PNS and CNS development.

This study aimed to analyze the association between median nerve CSA and HC by exploring the hypothesis that there is a close correlation between median nerve CSA and HC. To achieve this purpose, the median nerve in children aged between 0 and 3 years was visualized using high-resolution ultrasound. The CSA was then measured and compared with the children's HC.

## Methods

This cross-sectional study was conducted at the Children's Hospital of Eastern Switzerland between March 2023 and March 2024. It was approved by the local ethical committee and registered with the Swiss project database (EKOS BASEC reference number: 2019 to 02200 [Ethics Committee Eastern Switzerland Business Administration System for Ethics Committees]).

All children aged 0 to 3 years who were hospitalized in the Children's Hospital of Eastern Switzerland during the study period were eligible for inclusion in this study. Children with a neurological disease, a disease with possible neurological effects, or a chronic or severe acute illness were excluded from the study. Premature infants and former premature infants were also excluded. Most of the participants were hospitalized due to an infection and were examined shortly before leaving the hospital in an almost healthy condition. The caregivers of the participating children signed a written informed consent form before being enrolled in the study.

To control for variability between examiners, all examinations were conducted by one of two trained examiners: NB and LJ. Ultrasound imaging was conducted using an Aplio i800 system (Canon Medical Systems, Tokyo, Japan) equipped with an i22LH8 ultrasound probe with a maximum scanning frequency of 22 MHz. The general configuration, with an imaging depth of 17.5 mm, was employed, resulting in an axial resolution in the range of 50 µm.

**Fig. 1:**
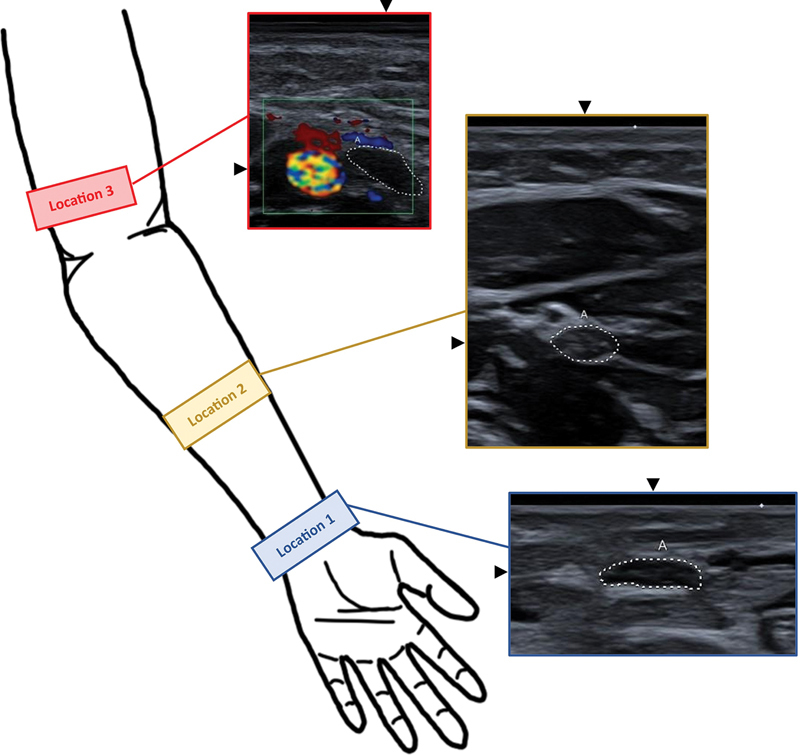
Schematic representation of locations 1, 2, and 3. Location 1 is on the wrist near the pronator quadratus muscle. Location 2 is on the forearm in the middle between the wrist and the elbow, in the middle of the forearm, where the median nerve runs between the superficial and deep flexor muscles. Location 3 is on the upper arm, directly above the elbow, where the median nerve runs with the brachial artery, which is depicted using color Doppler.

In accordance with Jenny et al, 14 median nerve images were taken at three locations on the arm ( Fig. 1 ). Location 1 was on the wrist at the level of the flexor retinaculum near the pronator quadratus muscle, where the nerve follows a characteristic course. Location 2 was half the distance between the wrist and the elbow in the middle of the forearm. In this position, the median nerve runs between the superficial and deep flexor muscles. Location 3 was on the lower third of the upper arm, just above the elbow, where the nerve runs in the immediate vicinity of the brachial artery. Doppler ultrasound was therefore used at location 3 to reliably distinguish the nerve from the artery. Although the aim was to examine both arms, this was not always possible due to medical equipment (e.g., intravenous lines) or a lack of compliance on the part of participants.

To better identify and measure the median nerve, transverse orthogonal still images and video recordings were taken at all three locations. To minimize the time each child needed to stay still, the images were saved and analyzed postexamination. To measure CSA in square millimeters and circumference in millimeters, the nerve was manually traced using the ultrasound system's freehand tracing tool. For all three locations, both values were stored in the study database.

Demographic and growth data, such as sex, age, height, weight, and HC, were collected from the participants' medical records and added to the study database. Height, weight, and HC percentiles relative to the population of Swiss Children were calculated and reported. 16

The HC values taken from the medical records were obtained from standardized measurements taken by the nursing staff. For this purpose, a nonstretchable measuring tape was placed around the largest part of the head, extending over the most prominent points of the skull and the center of the forehead. 17 The tape was tightened snugly to slightly compress the hair and the underlying soft tissue. To ensure accuracy, HC was measured twice. In the event of a discrepancy, the measurement process was repeated until the exact same value was obtained twice. Since the study was conducted by the same team and equipment, the methodology was similar to those used by Jenny et al 14 and Wurster et al. 18

All statistical analyses were performed using the statistical program R. 19 The package “ Table 1 ” 20 was used to compute the descriptive statistics, and the package “ggplot2” 21 was used to plot the figures. Since two reviewers were involved in the nerve measurement process, an intraclass correlation analysis was performed on the three area locations for four patients to ensure the reliability of the raters for the CSA of the nerves.

**Table 1:** Demographic and growth data of the participants ( *n* = 40)

Age (in mo)	
Mean (SD)	8.22 (8.83)
Median [min, max]	4.01 [0.0657, 33.7]
Sex	
Female	19 (47.5%)
Male	21 (52.5%)
Weight (kg)	
Mean (SD)	7.62 (3.48)
Median [min, max]	6.29 [2.63, 13.9]
Weight percentile	
Mean (SD)	49.0 (35.7)
Median [min, max]	47.8 [0.01, 99.2]
Height (cm)	
Mean (SD)	66.6 (13.6)
Median [MIN, MAX]	61.8 [46.5, 95.0]
Missing	2 (5.0%)
Height percentile	
Mean (SD)	50.9 (32.9)
Median [min, max]	52.9 [0.00, 100]
Missing	2 (5.0%)
Head circumference (cm)	
Mean (SD)	42.6 (5.02)
Median [min, max]	41.8 [33.5, 52.0]
Head circumference percentile	
Mean (SD)	57.0 (34.1)
Median [min, max]	61.6 [0.06, 100]

Abbreviations: *n* , number; SD, standard deviation.

Note: Shows an overview of the demographic and growth (height, weight, and head circumference) data of the participants, as well as their height, weight, and head circumference percentiles relative to the Swiss population.

Due to the presence of ties in the dataset, Kendall's correlation test was used to examine the statistical significance of the correlation between the three nerve CSA values and HC. In addition, linear regression models were performed to investigate the variables age and HC as potential predictors of CSA in unadjusted and adjusted models. The statistical significance level was set to 0.05, and the Benjamini–Hochberg procedure was performed to control for the multiple statistical tests in this study. 22 *p* -Values were ranked from smallest to largest and compared with their corresponding critical values calculated as (rank/total) × 0.05. This corrects for multiple statistical testing, though results should still be interpreted with caution, given the exploratory nature of the study.

## Results

This study included 40 participants (48% female) aged 2 days to 2 years and 10 months. The demographic data are listed in Table 1 , and their age and weight distributions are shown in Appendix A (Fig. A1, available in the online version only). Appendix B (Table B1, available in the online version only) shows the characteristics of the nerve CSA measurements for locations 1, 2, and 3.

### Size of CSA

**Fig. 2:**
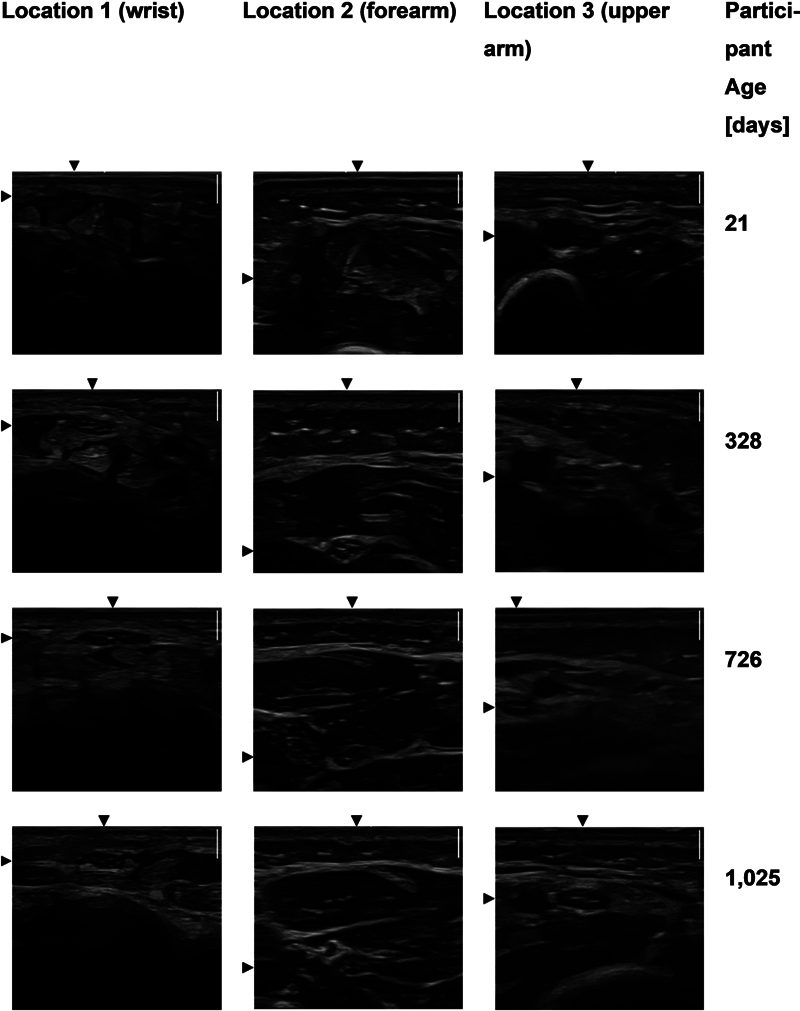
Examples of ultrasound images. This figure shows ultrasound images for locations 1, 2, and 3 for four participants of different ages (21–1,025 days). The white scale bar on the upper right side of each picture corresponds to 2 mm and applies to all of the following images. In each picture, the nerve is located at the intersection of the two arrowheads.

Fig. 2 shows exemplary ultrasound images of four participants sorted by location and age as they were used to measure the participants' CSAs. The measurements of the CSA were performed by two reviewers, and ICCs testing the reliability of the raters were all > 0.96, indicating high rater reliability ( Appendix B [Table B2, available in the online version only]). A purely visual increase in the size of the nerve with age can be seen in Fig. 2 , especially between the two youngest children. In Appendix A (Fig. A2, available in the online version only), CSA is plotted against age to better illustrate the growth process of the nerve.

### Head Growth

Appendix A (Fig. A3, available in the online version only) shows the relationship between HC and age. HC is an important anthropometric measure in pediatrics that typically increases with age and shows characteristic progression. The distributions and increases in CSA in Appendix A (Figs. A2, available in the online version only) and HC in Appendix A (Fig. A3, available in the online version only) with age were very similar, suggesting that an increase in median nerve CSA may be correlated and uniform with an increase in HC.

### Correlation Between CSA and HC

**Fig. 3:**
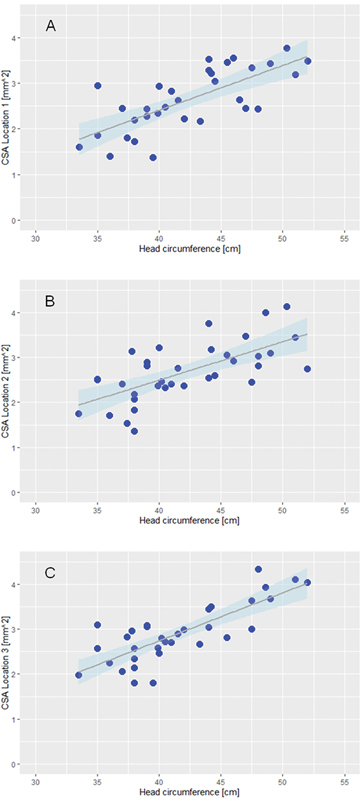
Scatterplots of the association between CSA and HC, including regression. The graphs show the association between the nerve cross-sectional areas at locations and head circumference, as well as the regression of the association, including its corresponding 95% confidence bands. ( **A** ) shows location 1, ( **B** ) location 2, and ( **C** ) location 3.

Fig. 3 shows the correlation between CSA for locations 1, 2, and 3, and HC. In the three scatterplots, HC is plotted in cm on the x-axis, and CSA is plotted in mm ^2^ on the y-axis. Each plot includes a linear regression line with its corresponding 95% confidence bands.

For each CSA location, unadjusted and adjusted models for the parameters age and HC were performed ( Tables 2 3 4 ). Since age and HC correlated strongly (Kendall's correlation = 0.80, *p* < 0.001), the effects of the interaction terms of age and HC on CSA were tested using linear regression models; however, since the interaction terms were not significant, these were left out of the models. The unadjusted models for all three locations showed that age and HC each had a significant influence on CSA. In the unadjusted models, for each month of age, the CSA increased by 0.46 to 0.57 mm ^2^ , and, for each cm increase in HC, the CSA increased by 0.09 to 0.11 mm ^2^ , for the three areas. When adjusting for both age and HC, age remained statistically significant only for area 3 (estimate: 0.03, 95% CI: 0.001–0.059), while HC remained significant for all three areas (area 1: estimate: 0.10, 95% CI: 0.028–0.168; area 2: 0.06, 95% CI: −0.009–0.119; area 3: 0.06, 95% CI: 0.005–0.113) suggesting that HC potentially better predicts CSA than age. While both age and HC showed significant associations in the unadjusted models, the effect sizes were generally smaller when analyzed in the adjusted models, suggesting multicollinearity between the two variables.

**Table 2:** Linear models of CSA with age and HC for location 1

Variable	Model estimate	95% CI	*p* -Value
Unadjusted models			
Age (in mo)	0.056	0.030–0.082	< 0.001
Head circumference (cm)	0.098	0.063–0.133	< 0.001
Adjusted models			
Age (in mo)	0.000	−0.046 to 0.046	0.999
Head circumference (cm)	0.098	0.028–0.168	0.008

Note: Show the unadjusted and adjusted linear regression models for the three locations of the CSA measurements as a function of age and HC.

**Table 3:** Linear models of CSA with age and HC for location 2

Variable	Model estimate	95% CI	*p* -Value
Unadjusted models			
Age (in mo)	0.046	0.027–0.065	< 0.001
Head circumference (cm)	0.085	0.052 0.119	< 0.001
Adjusted models			
Age (in mo)	0.020	−0.015 to 0.055	0.259
Head circumference (cm)	0.055	−0.009 to 0.119	0.090

Note: Show the unadjusted and adjusted linear regression models for the three locations of the CSA measurements as a function of age and HC.

**Table 4:** Linear models of CSA with age and HC for location 3

Variable	Model estimate	95% CI	*p* -Value
Unadjusted models			
Age (in mo)	0.057	0.041–0.073	<0.001
Head circumference (cm)	0.107	0.077–0.136	<0.001
Adjusted models			
Age (in mo)	0.030	0.001–0.059	0.041
Head circumference (cm)	0.059	0.005–0.113	0.032

Note: Show the unadjusted and adjusted linear regression models for the three locations of the CSA measurements as a function of age and HC.

The correction for multiple testing was performed using the Benjamini–Hochberg procedure to control the false discovery rate (FDR) at 0.05 across 15 *p* -values. Of the 15 *p* -values, 12 remained significant after FDR correction, with all tests having *p* -values at or below 0.041 maintaining statistical significance.

## Discussion

This study aimed to analyze the association between PNS and CNS development by using median nerve CSAs at three locations (wrist, forearm, and upper arm) and HC as proxies for PNS and CNS development, respectively. The study findings revealed a significant correlation between these proxies in children aged 2 days to 2 years and 10 months. Both the statistical analysis and the graphs in Fig. 3 indicated that there was a linear association between HC and CSA. The linear regression models showed that HC had a significant effect on CSA. This was evident in the unadjusted and adjusted models; the effects for locations 1 and 3 were lower in the adjusted models than in the unadjusted models, likely due to the multicollinearity between age and HC.

The findings supported the hypothesis that there is a close correlation between CNS and PNS development. These findings are consistent with the hypothesis that common developmental mechanisms may underlie central and peripheral growth, but this assumption requires further confirmation using longitudinal data. Cristobal and Lee 23 used animal models to show that similar molecular processes, such as the involvement of certain transcription factors and the protein-mediated regulation of actin dynamics in the CNS and the PNS, play crucial roles in central and peripheral myelination. Although speculative, these mechanisms are based on animal and in vitro studies and cannot be directly inferred from our findings.

In addition, a study by Trevers et al 24 has revealed commonalities between CNS and PNS progenitor cells during embryonic development, further supporting the hypothesis of synchronized development. The observed synchrony in CNS and PNS development can be attributed to several underlying mechanisms. Both the CNS and the PNS develop from the neural crest during embryogenesis, indicating a coordinated developmental process. 25

Olmsted and Paluh 26 used human gastruloid models to show that complex connections between the PNS and the CNS, such as the influence of the neural crest cells and chemical signaling pathways, play a role in the coordination of the two nervous systems. These and other connections promote the spatial and temporal synchronization of the two nervous systems by controlling their differentiation, pattern formation, and functional integration. Although the results of this study are consistent with studies that emphasized the intertwining of CNS and PNS development (23–26), an extensive literature search did not reveal any studies that directly investigated the temporal synchrony between HC growth and peripheral nerve development on the molecular level.

Among the study's strengths is its contribution to the scientific relevance and advancement of knowledge in the field of neurodevelopment. To our knowledge, this is one of the first studies to directly investigate the association between HC and median nerve CSA in infants. As such, this study fills an important research gap in understanding the association between PNS and CNS development.

The methodological accuracy of the study and the standardized implementation of measurements support the reliability of the results. The high interobserver reliability of the measurements underlined the precision of the data collection process.

Although this study provides valuable insights into the association between HC and median nerve CSA, it has several limitations that should be considered. First, the relatively small sample size of 40 participants limited the statistical power of the analyses and reduced the generalizability of the results. In addition, only a few children over 600 days of age were studied. The small population and the inclusion of only healthy term infants in the study limited the representativeness of the results, as they may not be transferable to preterm infants or infants with neurological or developmental abnormalities.

Second, the cross-sectional design of the study precluded the identification of causal relationships between HC and peripheral nerve development. Longitudinal data are needed for a more detailed investigation of a possible causal relationship. Although the observed correlation was intriguing, it could not definitively prove whether changes in HC drove the growth and development of the peripheral nerves, or vice versa.

Third, the study faced methodological challenges. Although the use of ultrasound measurements is practical and noninvasive, their implementation is highly dependent on the person carrying out the examination. In addition, nerve CSA is still very small at this stage of development, especially in children during the 1st months of life. This might have further complicated the measurement, and even minimal deviations could have led to relevant errors.

Fourth, this study only focused on one peripheral nerve—the median nerve—without examining other nerves. This limited the generalizability of the results to the entire PNS. Furthermore, no confounding variables, such as genetic factors, nutrition, or other growth parameters, were taken into account in the analyses. Such variables could have influenced HC and CSA and, therefore, affected the results.

Fifth, a major challenge in the regression analyses was the multicollinearity between HC and age. The high correlation between the two predictors (Kendall's correlation = 0.80, *p* < 0.001) potentially affected the regression coefficients and made it difficult to isolate their individual effects on CSA. Furthermore, 15 *p* -values were calculated, and while multiple comparisons were adjusted for, the results should still be interpreted with caution.

The study findings have important implications for research and clinical practice. One of the most important findings was the potential utility of HC as a marker for monitoring CNS and PNS growth and development. Given the observed association between HC and median nerve CSA, these parameters may represent interrelated aspects of neuronal development. This suggests that HC and CSA are not simply independent measurements but may reflect a common developmental process and may influence each other during critical growth phases.

HC has long been used in clinical settings as a noninvasive and easily measurable proxy parameter for brain growth and CNS development. The study findings demonstrate the possibility of integrating CSA measurements into routine clinical examinations to obtain a more comprehensive picture of neuronal development. Simultaneously measuring HC and CSA and comparing them could provide insights into the interplay of central and peripheral neurological development and allow clinicians to recognize deviations from typical growth patterns. This combined approach could prove particularly valuable for the early detection and intervention of conditions that affect neuronal development, such as congenital anomalies, growth disorders, and neurodegenerative diseases.

Furthermore, the findings highlight the need for a multidisciplinary perspective when assessing neuronal development. By linking anthropometric measures, such as HC, with structural parameters, such as CSA, future research could improve the understanding of how CNS maturation and PNS maturation are coordinated. This could lead to the development of new diagnostic tools or therapeutic strategies aimed at optimizing neuronal development during critical periods of growth.

To build on the results of this study, future research should address several key areas. First, studies with larger populations are needed to improve the statistical power and generalizability of the observed associations. Second, a longitudinal study design would allow for the exploration of growth trajectories as well as the dynamic correlation of HC and CSA over time. In addition, longitudinal data could further reinforce the assumption of a strong association between the parameters examined. This would provide valuable insights into previously unknown developmental patterns.

Future studies should include additional peripheral nerves and interdisciplinary perspectives to provide a broader understanding of PNS and CNS development. In particular, research involving preterm and neurologically impaired infants could clarify how gestational age and neurological conditions influence the HC–CSA association and whether abnormalities may serve as diagnostic markers. In this context, the findings of Jansen et al, 27 who demonstrated that median nerve maturation in preterm-born children parallels that of term-born peers, not only provide an important foundation for extending such investigations to populations at higher neurodevelopmental risk, but also underscore the potential of high-resolution nerve ultrasound as a feasible and clinically valuable diagnostic tool in this setting.

## Conclusion

The study findings support the hypothesis that the PNS development is closely correlated with HC, which is representative of CNS growth. The observed correlation between HC and median nerve CSA suggests an association, indicating parallel temporal patterns of growth in these systems. One of the key elements of the growth of the central and PNS is myelination. The association presented in this study suggests synchronous myelination in the CNS and PNS; however, this has to be proven in a longitudinal study.

Regarding the potential diagnostic relevance of the study findings, the parallel course of HC and CSA may serve as preliminary markers for the assessment of neuronal development. Deviations from typical growth patterns may indicate underlying neurological disorders and provide clinicians with a noninvasive tool for early detection and intervention. This possibility of applying the parameters has already been demonstrated in the work of Wurster et al 18 on spinal muscular atrophy (SMA). Children with SMA showed a significantly smaller CSA in the study compared with the control group, although there was no difference in HC between the two groups. Such findings are particularly valuable for the detection of developmental delays or abnormalities that may affect the coordinated growth of the CNS and the PNS.

This study also opens up new avenues for research and clinical applications. The integration of HC and CSA measurements into routine developmental monitoring could provide a more comprehensive view of neuronal development. These results could form the basis for future studies aimed at refining diagnostic tools and improving the general understanding of neuronal growth in typical and atypical populations.
