# Supplementary material for: Association Between Head Circumference Growth and Peripheral Nerve Cross-Sectional Area Growth in Infants: A Potential Future Biomarker for Central and Peripheral Nerve Maturation
Source: Neuropediatrics. 2025 Nov 28;57(2):131–8. doi: 10.1055/a-2747-7359 (PMC12956380; doi:10.1055/a-2747-7359)
Supplement: Supplementary file 1 — Supplementary Material [file 10-1055-a-2747-7359-s0320254017oa.pdf]

Supplementary Material

Appendix A, Figures

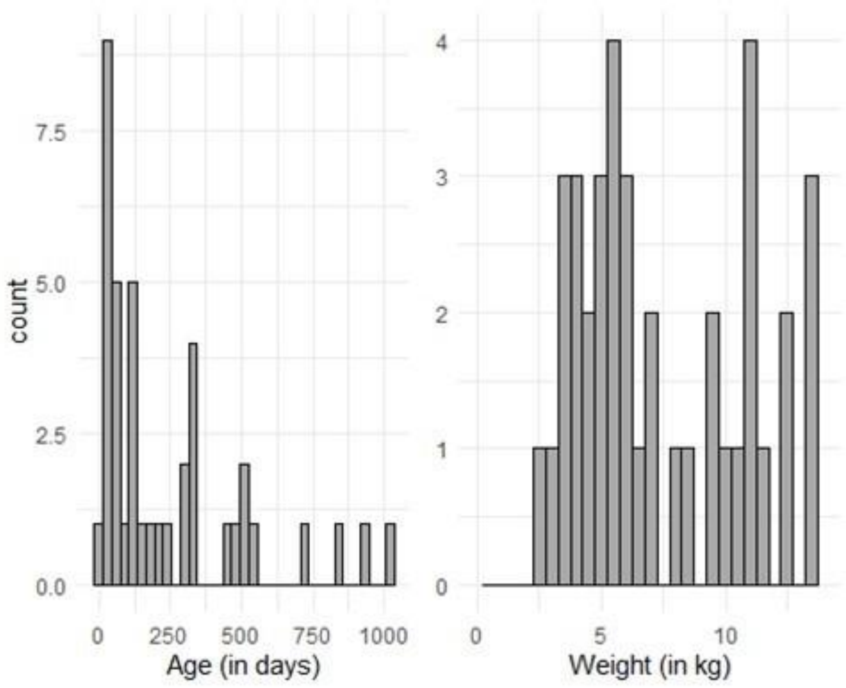

Figure A.1: Histograms of the age and weight of the participants

The distribution of the participants is shown in the histograms for age and weight.

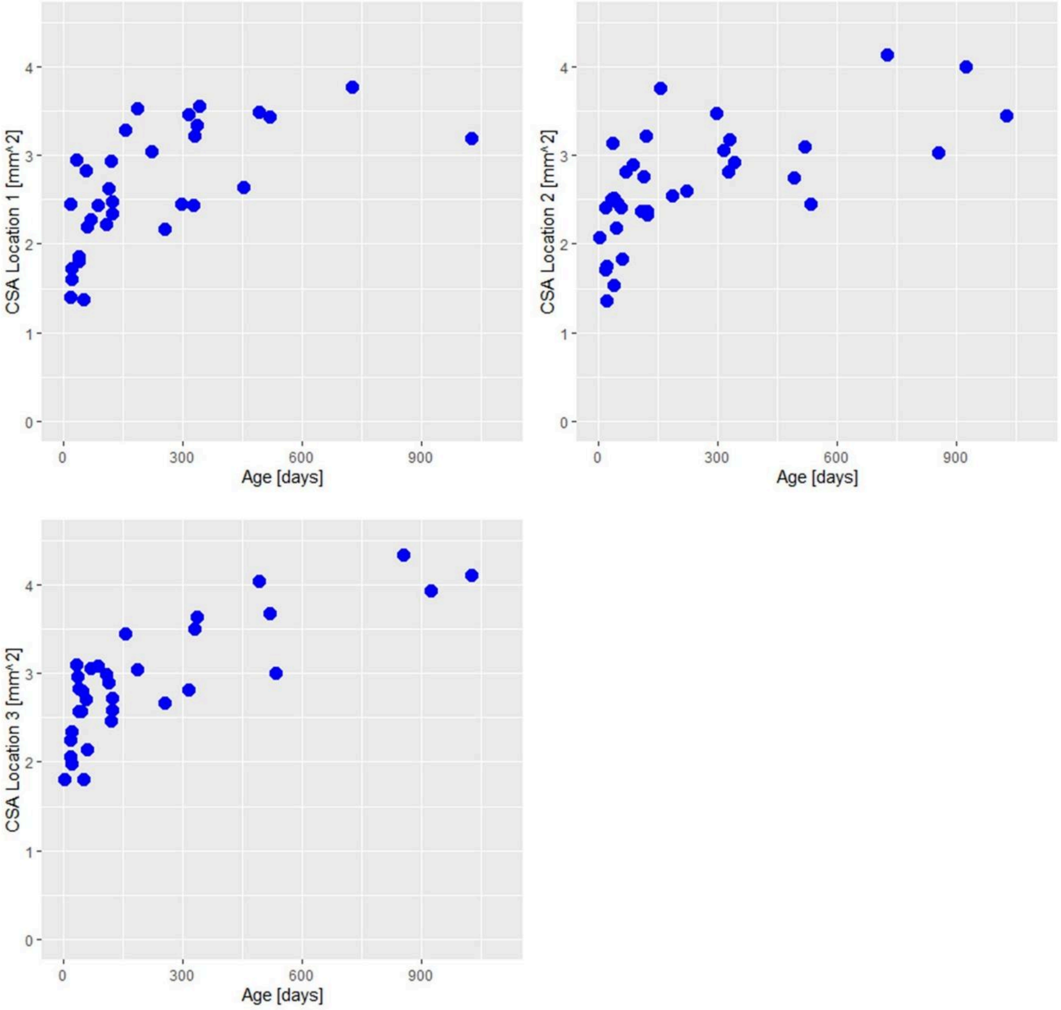

Figure A.2: Scatterplots of the relationship between CSA and age

The graphs show the nerve cross-sectional areas plotted against the age of the participants for location 1 (wrist), location 2 (forearm), and location 3 (upper arm).

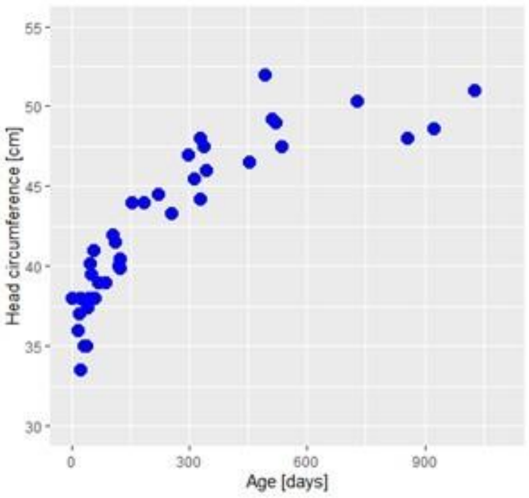

Figure A3: Scatterplot of the relationship between HC and Age

The figure shows the HC plotted against age.

Appendix B, Tables

Table B.1: Characteristics of the nerve CSA measurements at locations 1, 2, and 3

**Location 1**	
Mean (SD)	2.64 (0.676)
Median [Min, Max]	2.55 [1.37, 3.77]
Missing	8 (20.0%)
**Location 2**	
Mean (SD)	2.68 (0.650)
Median [Min, Max]	2.60 [1.36, 4.13]
Missing	5 (12.5%)
**Location 3**	
Mean (SD)	2.90 (0.654)
Median [Min, Max]	2.82 [1.80, 4.33]
Missing	7 (17.5%)

SD: standard deviation

Table B.2: Intraclass correlation analyses

Variable	ICC	95% CI	*p*-value	Sample Size
Location 1	0.996	0.938–1.000	< 0.001	4
Location 2	0.992	0.887–0.999	< 0.001	4
Location 3	0.964	0.563–0.998	0.004	4

This table shows the results of the intraclass correlation analysis performed by the two examiners.

ICC: intraclass correlation coefficient
